# Proceedings of the Michigan Central Dental Association

**Published:** 1871-11

**Authors:** 


### PROCEEDINGS OF THE MICHIGAN CENTRAL DENTAL ASSOCIATION.

A meeting of this body was held at Battle Creek on July 12th and 13th, at the parlors of the Potter House. Members were present from Olivet, Eaton Rapids, Kalamazoo, Lansing, Jackson, Marshall, Grand Rapids, Ann Arbor, and other towns throughout the State.

Officers:

*President*—Dr. D. C. Hawxhurst.

*Vice President*—Dr. L. C. Whiting.

*Secretary*—Dr. H. P. Peterson.

*Corresponding Secretary*—Dr. Geo. H. Cole.

*Treasurer*—Dr. T. A. White.

After some general business, the regular discussions of the society were opened by an essay from Dr. Geo. P. Holmes, on "Professional Ethics." He thought that every dentist should array himself in the garb of true gentlemanliness in which to meet his patients. He should also exhibit the most scrupulous courtesy in his dealings with his professional brethren. But even these shining qualities are of comparative insignificance without a basis of true integrity. Possessed of these qualifications and the requisite energy, his road to success would be of easy access. The "Dr." and "M. D." upon cards and signs are of little value. Society has so often been cheated by them, that he who flourishes the most, ever making them conspicuous, is looked upon with suspicion. The prevalent system of advertising was productive of much harm. Cards, handbills, posters and show case advertisements, calling attention to the "unsurpassed facilities," the "extensive experience" of the undersigned to produce a "peculiar style of work and cheaper than any in town," savors strongly of quackery and should be disapproved by the association. He recommended the adoption of the "Code of Ethics" of the American Dental Association. It was a high toned professional production, a copy of which should be hung in the office of every dentist.

He approved of such a form of advertising as would be instructive to the people in whatever form it should be gotten up. He hoped the Association would adopt a Code of Ethics.

A long discussion followed. Dr. Smith was opposed to that part of the Code of Ethics (recommended by the essayist) which prohibited advertising. Thought our association was a primary school and ought not to be so strict with its members as the national association. We must be content with the gradual progress of our members, and not expect them to make the whole distance at a leap. Besides, advertising is right, if done properly. All excellence should make itself known. All legitimate business succeeds by advertising. People expect it. If a man can do something well, why not allow it to be known? But a style of glaring quackery need not be adopted. The standard of the profession should be dignified. Without this titles and degrees are nothing. If a man is introduced into our association he is our brother, provided the examining committee have done their duty. If he violates the principles of professional dignity and right practice, let us report him.

Dr. Cole moved the adoption of the Code of Ethics. Thought that there were other ways of advertising except by cards, handbills, etc. Every dentist could write articles for his local paper that would instruct the people, give him wide notice, and at the same time, furnish some evidence of his abilities and real qualifications. The subject matter should be exclusively scientific.

Dr. Fuller thought perfect works the best advertisement.

The discussion was long and animated. Most of the members participated. We have given enough to indicate the general character of this session, which was closed by the adoption of the Code of Ethics.

### SECOND SESSION.

Dr. Cole opened with an essay on "Inspiration through the Mouth." Said a beat of the heart and a breath of air are all that separate us from death. How important, then, that this breath be free from foreign matter. Prof. Tyndall has shown that in iron factory air there are minute particles of carbon, ash, and iron; in shirt factory air are microscopic filaments of linen and cotton; in air of flouring mills and oat mills are fibrous fragments, starchy grain and spores; antimony is found in air of printing offices and in type foundries Dirty air, entering the lungs, must irritate the delicate membranous surfaces. Prof. Tyndall further discovered that of all the dust constantly entering the lungs, none was thrown off. Are we to conclude that it remains in the system? Respiration through the nose will strain the air of these atoms. The extensive moist surfaces of the schneiderian membrane catch the floating atoms and absorb odors. In treating patients who have bad breath, the practitioner should breathe through the nose. Colds often result from taking the frosty air of winter directly into the lungs without a preparatory warming through the devious corrugations of the nasal passages.

The essay was discussed as follows: Dr. Holmes thought the question important. The nose is a filter; it is the natural purifier of the inspired air; especially when working over decayed teeth we should breathe through it. Some patients have breath that is simply disgusting; we should not work for them until some means is taken to make the breath sweet. That source of bad breath which is most difficult of treatment is catarrh—a peculiarity of this country.

Dr. Smith thought breathing through the nose not always advisable. In nasal catarrh the inspired air thus becomes ladened with heavy vapors, injurious to health. Would it not in such cases be better to breathe through the mouth, and get a purer air?

Dr. Cole could not agree with Dr. Smith. If the nose isn't fit to breathe through, cleanse it, wash it out, and make it fit to breathe through. Nothing but harm can come from a disuse of the nose. Like any other neglected part, it rapidly deteriorates, its passages diminish, its membranes dry up. The nose must be used in catarrh, even if you have to rinse the entire passages hourly.

Dr. Way, President of the Northern Ohio Dental Association, thought the breath of a catarrhal patient worse than any other; thought it the most common cause of bad breath; considered it very exhausting to work for such patients, and certainly very unpleasant. With reference to bad taste, which had been so much talked about, thought it due to drying or evaporation of saliva, except where the mouth was not cleansed well with a brush; in such cases the mouth might be foul from ferment of fragments of food. People do not well clean their teeth. The brush inserted, a few motions made, and the mouth is thought to be clean. Thought if we were called upon to choose between tooth pick and brush, we would choose the former, though we would prefer both. The tooth pick reaches between the teeth best.

Dr. Hawxhurst asked Dr. Smith to take the chair while he responded to a call from the house. Would direct attention to the cotton respirator, as a protection against bad breath. Its fine meshes strain off and absorb bad odors from the patient. It divides the inspired air into a myriad of fine streams, which flow over the dampened fibres of cotton, which entangle and keep back particles of floating dirt. It should be often cleansed. Regards the nose as the natural respirator. By the corrugation of its extensive surface, as well as the moisture of its membranes, it deodorizes foul air. Foul atoms are promptly caught in its moist and devious passages. It is a delicate instrument, and must be kept in good order. Fumes and gases from an overloaded stomach may dry up its passages and make it little better than a tin pipe to breathe through. It will neither keep foul odors back from the lungs, nor assure its possessor that his person and office are not offensive to patients. Thinks a good nose of more importance than deodorizers, since it teaches when to use them.

### THIRD SESSION.

Discussions opened on the subject: "Morbid Conditions of the Mouth and their Treatment." Was participated in by Drs. Bartlett, Jackson, Robinson, Smith, Cole, Penniman, Holmes, and others. The technicalities of medicine, chemical nomenclature and the language of pathology reigned supreme through this discussion, and we pass to other matters of greater interest to the public.

Following this was an essay by Dr. Peterson, on Nasal Catarrh, which was too learned and scientific for general reading. While speaking of the causes he gave some cases in which neglected roots had induced nasal catarrh. Thought that diseased molars, whose roots extend near the antrum, are liable to aggravate it. Referred to a case which was cured by washes of dilute permanganate of potash. Advised homœopathic treatment.

A lively discussion followed, during which Dr. Robinson related a case of extreme bad breath from catarrh. He advised the simple douche of salt and water. It not only gave immediate relief, but effected a permanent cure. Considered it best given by aid of a rubber-bag syringe, kept by druggists.

Dr. Jackson read an essay on "The Best Preparation of Gold for Filling Teeth." The Doctor thought there was much difference of opinion among dentists on this subject. Whatever the form used, much depends on the mode of working it. What with one would be a failure, with another would be a success. Most operators have fallen into some one method of filling, and can use that form of gold best to which it is adapted. Some operators are intelligent enough to work each form of gold by its own method. Such are successful with all, and use all, at times. There is no *best* form of gold. Then followed an exceedingly able and discriminating description of each preparation and the cases in which it should be used. The essay being finished, a long discussion followed, by Drs. Way, Jackson, Smith, Robinson, Bartlett, and numerous others. The matter was too technical to be of interest to the public.

Dr. Hawxhurst gave a plan for furnishing the association with instruction. He regarded it of great importance that we keep ourselves informed on the subject of improved methods of practice. A whole army of ingenious intellects are constantly devising better modes, inventing better instruments, and discovering new principles. Unless we wish to be left in the rear, we must possess these improvements. Thought we ought to send for such men as Taft, Allport, Watt, Atkinson and Palmer, and pay them for lecturing to our association; would recommend Dr. Corydon Palmer, to assist us at our next meeting. Drs. Way and Robinson spoke in high commendation of Dr. Palmer, and favored the plan.

The following motion was carried by vote: That Dr. Corydon Palmer, of Warren, Ohio, be requested to give a two days' course of "Demonstrations on Practice," before the Michigan Central Dental Association at its next meeting."

Another motion was passed, empowering the Corresponding Secretary to arrange terms with Dr. Palmer.

Dr Hawxhurst read a long essay on "The Relation of the Microscope to Dental Practice." It was well written, but too technical for public reading. It was received with strong marks of favor by the association.

Another motion was adopted, as follows: That the house appoint a committee on general interests of the association, whose duty it shall be to investigate the subject of publishing association reports; to observe and report the advertising practices of members; to suggest methods of instruction for the association; to enlarge the membership; to study how to make the meetings of the association interesting; and to oversee its general interests.

Drs. Hawxhurst, Robinson and Penniman were appointed.

It was moved and carried that the next meeting be held at Jackson, on the second Wednesday of January, 1872.

A vote of thanks was tendered to the proprietor of the Potter House of this city, for his courtesy in opening his parlors for the meetings of the association, free of charge.

Meeting adjourned.

